# Peripheral nerve injury reduces the excitation-inhibition balance of basolateral amygdala inputs to prelimbic pyramidal neurons projecting to the periaqueductal gray

**DOI:** 10.1186/s13041-020-00638-w

**Published:** 2020-06-29

**Authors:** John Cheriyan, Patrick L. Sheets

**Affiliations:** 1 Department of Pharmacology and Toxicology, Indianapolis, IN USA; 2 Stark Neurosciences Research Institute, Indianapolis, IN USA; 3 present address: Indiana Center for Biomedical Innovation and Department of Anesthesia, Indiana University School of Medicine, Neuroscience Research Building 400 D, 320 West 15th St, Indianapolis, IN 46202 USA

## Abstract

Cellular and synaptic mechanisms underlying how chronic pain induces maladaptive alterations to local circuits in the medial prefrontal cortex (mPFC), while emerging, remain unresolved. Consistent evidence shows that chronic pain attenuates activity in the prelimbic (PL) cortex, a mPFC subregion. This reduced activity is thought to be driven by increased inhibitory tone within PL circuits. Enhanced input from the basolateral amygdala (BLA) to inhibitory neurons in PL cortex is one well-received mechanism for this circuit change. In mice, we used retrograde labeling, brain slice recordings, and optogenetics to selectively stimulate and record ascending BLA inputs onto PL neurons that send projections to the periaqueductal gray (PAG), which is a midbrain structure that plays a significant role in endogenous analgesia. Activating BLA projections evoked both excitatory and inhibitory currents in cortico-PAG (CP) neurons, as we have shown previously. We measured changes to the ratio of BLA-evoked excitatory to inhibitory currents in the spared nerve injury (SNI) model of neuropathic pain. Our analysis reveals a reduced excitation-inhibition (E/I) ratio of BLA inputs to PL-CP neurons 7 days after SNI. The E/I ratio of BLA inputs to CP neurons in neighboring infralimbic (IL) cortex was unchanged in SNI animals. Collectively, this study reveals that the overall E/I balance of BLA inputs to PL neurons projecting to the PAG is reduced in a robust neuropathic pain model. Overall, our findings provide new mechanistic insight into how nerve injury produces dysfunction in PL circuits connected to structures involved in pain modulation.

Circuit and cellular dysfunction in the medial prefrontal cortex (mPFC) has been well-documented in rodent models of chronic pain [1–9]. Extensive evidence indicates that alterations to long-term plasticity of synaptic inputs within the mPFC contributes to chronic pain states [10]. Both arthritic and neuropathic pain models augment basolateral amygdala (BLA) inputs that drive feed-forward inhibition of layer 5 (L5) pyramidal neurons in mPFC [3, 5]. A major target of L5 neurons in mPFC is the periaqueductal gray (PAG), which is a key midbrain structure involved in descending inhibition of ascending nociceptive inputs [11, 12]. Additional work reports that nerve injury enhances inhibition of mPFC output to the PAG [4]. Here we aimed to determine whether the spared-nerve injury (SNI) model of neuropathic pain attenuates the ratio of excitatory to inhibitory (E/I) currents in cortico-PAG (CP) neurons in mPFC following optogenetic excitation of BLA axons in acute brain slice.

As we’ve done previously in our lab [13], we injected Alexa Flour-647 conjugated Cholera toxin B (Thermo-Fisher; ~ 100 nL) into the PAG and AAV1.CAG.ChR2-Venus.WPRE.SV40 (Addgene 20,071, ~ 100–200 nL) into the BLA of male or female C57BL6/J mice (stock# 00664; Jackson Labs; Fig. 1a). Mice were allowed to recover for at least 7 days before introducing the spared nerve injury (SNI) that involved removing ~ 2 mm sections of the peroneal and tibial nerve while leaving the sural nerve intact (Fig. 1b). Seven to 8 days following surgery, animals in the SNI group displayed robust mechanical allodynia while sham surgery animals showed no change in pain behavior (Fig. 1c). Following behavioral analysis, mice were briefly anesthetized (15–20 s) with isoflurane, decapitated and brains were rapidly removed. Coronal sections (300 μm) of the mPFC were prepared for optogenetic and electrophysiological recordings and clearly showed overlap of BLA axons and retrogradely labeled CP somata (Fig. 1d). Fluorescent retrogradely labeled CP neurons were selectively visualized for this study using a far red filter (660 nm emission) in line with a coolLED system (Scientifica, UK). Broad field excitation of ChR2+ BLA axons was performed using a 470 nm LED stimulus through the same coolLED system at ~ 40 mW output to the slice for three milliseconds. Series resistance was required to be < 35 MΩ and to have ≤15% variation between the initial and final reading to be included in the analysis. Recordings were filtered at 2 kHz and digitized at 10 kHz. Excitatory (EPSC) and inhibitory (IPSC) responses to photoactivation of ChR2+ BLA projections were recorded at command voltages of − 70 and + 10 mV, respectively in the presence of NMDA receptor antagonist CPP (5 μM, Tocris, UK). Data analysis was performed offline using Matlab routines (Mathworks, Inc.).

**Fig. 1:**
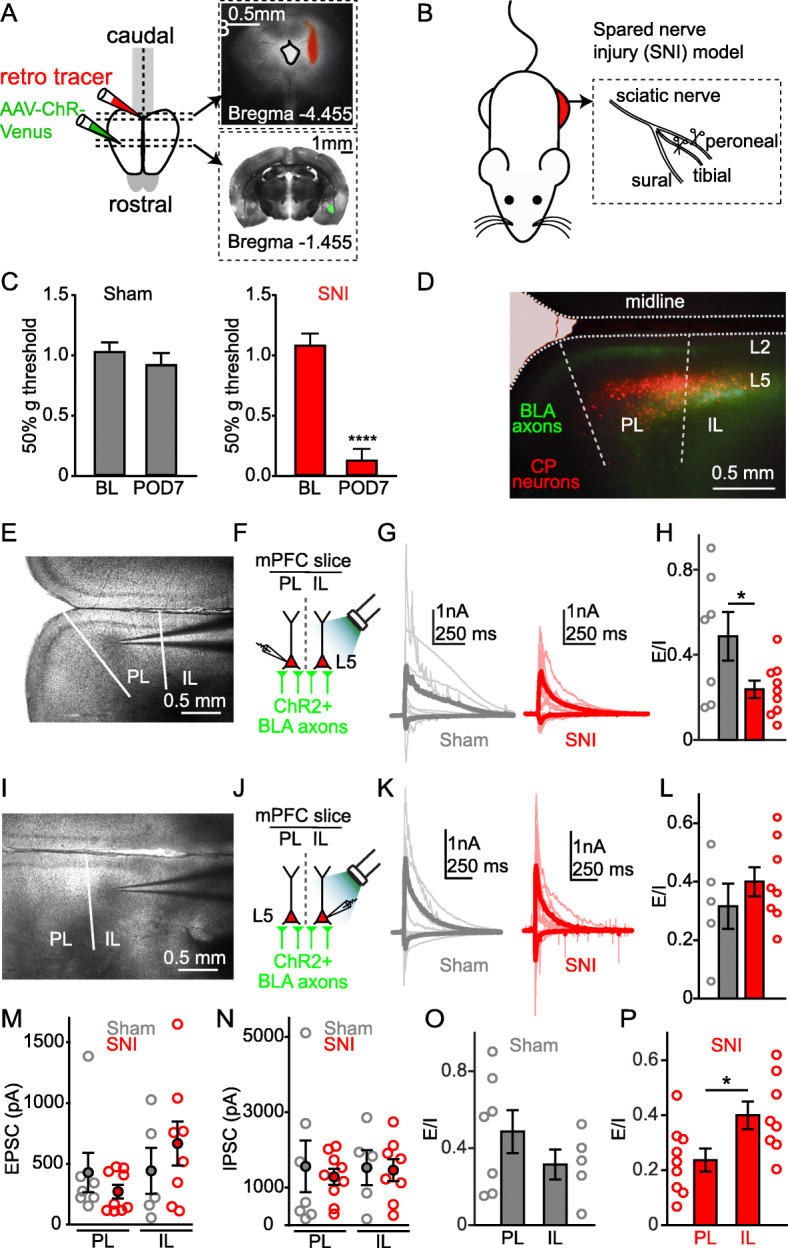
Nerve injury alters excitation-inhibition balance of inputs to cortic-PAG neurons in mPFC

Whole-cell slice recordings from layer 5 (L5) CP neurons in PL cortex contralateral to sham/SNI revealed that the ratio of peak excitatory input to peak inhibitory input (E/I ratio) evoked by optogenetic stimulation of ChR2+ BLA axons was significantly lower (t _(14)_ = 2.3, *p* = 0.04, Student’s unpaired t-test) in SNI animals (Fig. 1e-h). However, this decrease in E/I ratio of BLA inputs was not detected (t _(11)_ = 0.96, *p* = 0.36, Student’s unpaired t-test) in recordings from L5 CP neurons in IL cortex, which is ventrally adjacent to PL cortex (Fig. 1i-l). As in our previous work [11], we distinguished IL from PL by differences in cytoarchitecture including a distinguishable narrowing of layer 3 and an infiltration of layer 2 cells into layer 1 of IL. Peak amplitudes for EPSCs and IPSCs did not differ in PL-CP and IL-CP neurons recorded from sham and SNI mice (Fig. 1m, n). There was no statistical difference (t _(10)_ = 1.14, *p* = 0.28, Student’s unpaired t-test) in E/I ratios between PL-CP and IL-CP neurons recorded from sham mice (Fig. 1o). However, in SNI mice, the E/I ratio recorded from PL-CP neurons was significantly reduced compared to IL-CP neurons (t _(15)_ = 2.53, *p* = 0.02, Student’s unpaired t-test; Fig. 1p). While we did not observe sex dependent effects, further experiments are needed to substantiate this observation.

We have shown that BLA disynaptically drives inhibitory inputs to CP neurons via activation of local GABAergic neurons in both PL and IL cortex [13]. Previous work has shown that the BLA preferentially targets parvalbumin-expressing inhibitory neurons (PVINs) in mPFC over pyramidal neurons and other inhibitory neuron subtypes [14]. Following SNI, BLA inputs onto PVINs in PL cortex are augmented due to reduced presynaptic endocannabinoid signaling [4]. These findings align with our current data showing that SNI reduces the E/I ratio of BLA-driven inputs on CP neurons in PL cortex. This reduction in E/I ratio is likely driven by an enhancement of local inhibitory inputs from PVINs onto PL-CP neurons thereby reducing output to the PAG, which has been shown to contribute to neuropathic pain [4]. Activating the PL-PAG pathway may be a novel therapeutic strategy for treating pain as optogenetic stimulation of PL axons in the PAG has been shown to alleviate pain behavior in SNI mice [4]. Indeed this may be feasible as low-frequency electrical stimulation of the PL cortex has been shown to reduce aversive responses in a persistent inflammatory model [15], which likely involves increasing the activity of the PL-PAG pathway.

We have previously shown that SNI reduces excitability of CP neurons in PL, but not IL, cortex [1], which is consistent with our data showing no change to the E/I ratio of BLA-driven inputs onto IL-CP neurons. This dichotomy could be because BLA neurons targeting the PL and IL are distinct populations [16]. This raises the possibility that BLA neurons targeting IL do not express presynaptic endocannabinoid receptors, but this still needs to be elucidated. Nonetheless, SNI drives a separation in E/I balance between CP neurons in PL and IL.

Overall, our data build upon an emerging mechanism for how nerve injury drives dysfunction in cortical circuits upstream of structures essential for endogenous analgesia. While these circuits are likely critical for immediate top-down control of pain, it is becoming clearer that maladaptive changes to these same circuits may be a significant contributing factor to neuropathic pain following nerve injury.

**A)** Schematic and bright-field images of retrograde tracer injection into periaqueductal gray (PAG) and AAV-ChR2-venus virus injection into basolateral amygdala (BLA). **B)** Diagrammatic representation of spared nerve injury (SNI) model of neuropathic pain in the mouse. **C)** Mechanical allodynia measured using von Frey test in sham (F_(1,20)_ = 1.24; *p* = 0.28) and SNI (F _(1, 13)_ = 28.2; *p* < 0.0001; Two-way ANOVA) animals at baseline and post-operative day (POD) 7 or 8. **D)** Representative image of ChR2+ BLA axons (green) retrogradely-labeled cortico-PAG (CP) neurons (red) in coronal section of the prelimbic (PL) and infralimbic (IL) regions of medial prefrontal cortex (mPFC). **E)** Representative (4x) image showing recording from a CP neuron in PL cortex. **F)** Schematic showing recording configuration for measuring synaptic currents in PL-CP neurons following stimulation (470 nm, 3 milliseconds) of ChR2+ BLA axons. **G)** Overlay of total excitatory and inhibitory post-synaptic currents from PL-CP neurons from sham (grey) and SNI (red) mice. Thick line trace is the average of individual traces. **H)** Ratio of excitatory and inhibitory (E/I) inputs to PL-CP neurons from sham and SNI mice (*p < 0.05; Student’s unpaired t-test). **I)** Representative (4x) image showing recording from a CP neuron in IL cortex. **J)** Schematic showing recording configuration for measuring BLA input to IL-CP neurons. **K)** Overlay of total excitatory and inhibitory post-synaptic currents from IL-CP neurons. Thick line trace is the average of individual traces. **L)** Ratio of excitatory and inhibitory inputs to IL-CP neurons. **M)** Peak EPSC values (solid circle: mean ± SEM) for PL-CP (sham: 428 ± 162.5; SNI: 271.3 ± 56; t _(14)_ = 1.01, *p* = 0.3305) and IL-CP (sham: 441 ± 188.2; SNI: 665 ± 180; t _(11)_ = 0.82, *p* = 0.43, Student’s unpaired t-test) neurons. **N)** Peak IPSC values for PL-CP (sham: 1562.4 ± 685.5; SNI: 1284.2 ± 214; t _(14)_ = 0.43, *p* = 0.67) and IL-CP (sham = 1531 ± 465.3; SNI: 1462.6 ± 293; t _(11)_ = 0.13, *p* = 0.9, Student’s unpaired t-test) neurons. **O, P)** E/I ratio comparisons of PL and IL cortex from (**O**) sham and (**P**) SNI mice (*p < 0.05; Student’s unpaired t-test).

## Data Availability

All data generated or analyzed during this study are included in this published article. Data files used for this manuscript are available via a direct and reasonable request to the corresponding author and approval from Indiana University.
